# Supplementary material for: Are carbon emissions trading and green financial instruments synergistic? -Comprehensive quantitative research based on content analysis
Source: PLoS One. 2024 Mar 7;19(3):e0298601. doi: 10.1371/journal.pone.0298601 (PMC10919601; doi:10.1371/journal.pone.0298601)
Supplement: S1 Data — (ZIP) [file pone.0298601.s001.zip › Data/地级市Z_F工作报告.zip!/╣π╢1⁄2/╟σ╘╢╩╨/└┤╫╘╣1⁄2╓┌║┼í╛┬φ┐╦╩2╛▌═°í┐_2016.docx]

各位代表：

我代表市人民政府向大会作工作报告，请予审议，并请政协各位委员和其他列席人员提出意见。

2015年发展取得新成效 “十二五”目标任务较好完成

2015年是我市经济稳增长的关键一年，也是转型升级取得有效突破的一年。一年来，面对经济下行压力不断加大的严峻形势，在省委、省政府和市委的正确领导下，在市人大及其常委会和市政协的监督支持下，市政府全面贯彻党的十八届三中、四中、五中全会和习近平总书记系列重要讲话精神，紧紧围绕“南融北康”发展目标，积极适应经济新常态，大力推进广清一体化，着力稳增长、调结构、促改革、惠民生，较好完成市六届人大五次会议确定的目标任务，发展呈现新亮点：清远高新区晋升国家级，广清一体化战略上升到省级层面，千亿元重大项目全面动工，农村综合改革取得重大进展，金融机构贷款突破千亿大关，我市发展后劲位居全省前列。一年来，我们突出抓好八项工作：

（一）突出抓好稳增长，经济发展有新成效。出台扶持工业企业、外经贸和房地产行业稳增长政策措施，经济企稳回升势头进一步巩固。全市完成生产总值1285亿元，比上年增长（以下简称增长）8.4%，比全省和上年分别快0.4和0.5个百分点。完成固定资产投资620.6亿元，增长4.1%；一般公共预算收入108.4亿元，增长2.8%；社会消费品零售总额570.5亿元，增长9.7%；农林牧渔业总产值293.9亿元，增长4.5%；外贸出口168.7亿元，增长14.9%。接待旅游总人数3318.8万人次，旅游总收入241亿元，分别增长5.3%、10.3%。商品房销售面积、销售额分别增长29.1%和24.1%。坚定不移扩投资促增长，省、市重点项目完成投资340.5亿元，占年度投资计划的119.4%，完成率比上年提高16.3个百分点。其中，省重点项目完成年度投资计划的134.3%，完成率上升到全省第三位。积极加大融资力度，与金融机构达成意向合作金额近240亿元，新设立清远市特色农业担保基金和清远市电子商务发展担保基金，加快组建中小微企业融资政策性担保公司。争取省发债转贷资金支持，获得地方政府债券38.2亿元；棚户区改造、粤东西北振兴发展、省级职教基地建设等项目获得上级支持资金44亿元。金融机构本外币存款余额1699.8亿元、贷款余额1061.2亿元，分别增长10.8%和11.3%。2个PPP项目列入省示范性项目。1家企业在香港交易所上市，3家企业在“新三板”挂牌，实现我市企业新三板融资“零突破”。

（二）突出抓好新兴产业，转型升级有新步伐。一批投资达千亿元的产业项目成功落地。其中，总投资300亿元的长隆森林主题公园全面动工，动植物种源基地建设顺利推进。神华国华清远电厂首期两台百万千瓦“近零排放”机组正式动工。新兴际华、通用电梯、广百海元物流、万达广场等重大项目也已全面动工。全市新引进工业项目129个，合同投资额474.2亿元。新批设立外商投资项目17个，合同外资金额2.4亿美元，实际吸收外资金额1.4亿美元。全力实施工业转型升级攻坚战三年行动计划，完成工业技改投资32.8亿元，增长114.8%，增幅居全省第一。大力推进科技创新，全市专利申请受理量1569件，其中发明专利申请受理量346件，分别增长77.9%和116.2%，增幅均居全省第一；专利授权量1017件，其中发明专利授权量110件，分别增长61.4%和111.5%；组建大数据中心，与腾讯、苏宁、铁塔、电信、联通等公司合作，加快电商、智慧城市和大数据建设。

（三）突出抓好广清一体化，“南融北康”有新进展。推动广清一体化战略纳入省“十三五”规划。出台南部地区加快融入珠三角行动计划，着力推进交通、产业、公共服务、要素配置、城市功能、生态环境等六个方面的一体化发展。广清城轨清远段累计完成总投资的47%；佛清从高速北段、广清与清连高速连接线、汕湛和汕昆高速清远段、北江航道扩能升级项目全线动工；启动“五位一体”综合交通枢纽规划设计。147所学校、12家县级以上医院与广州建立帮扶关系，广清住房公积金实现互贷，金融、农业、旅游对接不断深化。出台北部地区全面建成小康社会行动计划。全面落实扶持民族地区发展有关政策。2015年省、市下达北部四县（市）财政转移支付资金77亿元，增长62.7%；下达财政配套资金2.4亿元，增长52.5%。加快北部地区交通、水利基础设施建设，连佛高速、韶柳铁路、韶贺高速前期工作顺利推进。加快贫困地区脱贫奔康步伐，284个重点帮扶村累计投入帮扶资金26.2亿元，重点帮扶村有劳动力的贫困户人均纯收入增长3.9倍，圆满完成第二轮扶贫“双到”工作任务。

（四）突出抓好重点园区，平台建设有新突破。大力实施以升促建，清远高新区成为全省第十一个、粤东西北第二个国家级高新区。天安智谷展示中心和运营中心建成投入使用，62家科技型中小企业签订入园协议；华南863科技创新园一期建成投入运营，入园项目47个；国家火炬清远高性能结构材料特色产业基地获得批准；以“政产学研金”合作方式，共建新材料中试加速基地和新材料协同创新研究院。“广州北”大学生创新创业基地、高新区创客中心顺利推进。重点产业平台建设取得新突破。广清产业园“三纵三横”路网基本形成，16项基础设施配套项目有序推进；已签约入园项目58个，计划总投资327亿元；在谈项目40多个；富强、迅通、欧派等35个项目正式动工，超额完成省定任务。“两德”合作区4条主干道路基本建成，投产企业6家，中南片区开发正式启动。4个省级园区新开工项目78个，在建项目156个；完成企业固定资产投资123亿元，实现规上工业增加值113.2亿元、税收21.7亿元，分别增长21.7%、29.3%、24%。5个产业集聚地获省认可，华南声谷（互联网+创新产业园）建设全面铺开。

（五）突出抓好环境治理，生态建设有新成果。全面启动国家环境保护模范城市创建工作。成立环境保护委员会，设立环境与食品药品犯罪侦查支队，成为全省第三个、粤东西北首个设立“环保警察”的地级市。理顺市、区环保执法事权关系。建成洲心、告星、望埠等3座污水处理厂。整治规模化畜禽养殖场599家，完成12家纺织、造纸企业废水处理设施和2家玻璃企业脱硫设施建设。取缔老旧游船111艘。淘汰黄标车1.87万台，扩大市区“黄标车”限行区域和高污染燃料禁燃区。完成大气预警预报平台建设，全面淘汰小锅炉、小冶炼，实施散体物料密闭运输和陶瓷瓷土集中开采配送。全面取缔关闭龙塘、石角园区外的拆解散户，完成重金属生态修复首期工程，启动电子废弃物整治二、三期工程建设。深入开展全国文明城市、国家卫生城市创建活动，实施“门前三包”责任制，城市环境进一步改善。全市森林覆盖率提高到 72%。空气质量明显改善，其中12月份居全省第一。主要河流水质稳定维持在良好以上水平。

（六）突出抓好农综改革，农业农村有新面貌。认真落实农村综合改革各项工作，深入推进“三个重心”下移，完善乡村治理机制，农村组织化水平和内生发展动力进一步提高。探索推进“三个整合”，全市共整合耕地134.4万亩，整合涉农资金12.5亿元，成立农村集体经济组织20679个。乡镇农村产权流转管理服务平台全面建成，完成土地流转60.8万亩，土地确权颁证6191户。村一级金融服务站实现全覆盖。启动信用镇、信用村、信用户评定工作，发放“政银保”贷款约3.6亿元。积极推进连州市和阳山县省级新农村连片示范建设工程。204条村庄通过年度美丽乡村考核验收。共建成7个农村电商县级运营中心、180个村级服务站，建成全省覆盖面最广的农村电商服务体系。

（七）突出抓好民生实事，社会事业有新进步。全市民生投入219.3亿元，增长39.5%，占一般公共预算支出的74.8%。城镇居民人均可支配收入、农村居民人均纯收入分别增长8.4%和9.8%。新增城镇就业5.12万人，城镇登记失业率2.37%。大幅提高底线民生和教育经费补助标准；完成633公里国省道路面改造、500公里农村公路硬底化建设和100座县乡公路危桥维修加固改造工程；15宗中央项目、5宗中小河流治理省试点项目以及17宗非试点项目完成年度建设任务；保障性住房开工4677套，公租房建成583套，新增低收入住房保障家庭租赁补贴998户；完成146个公交候车亭和556个公交站牌建设，市区新增公交车90辆；建立了系统全面的水质监控体系，水质监测水平进一步提升；完成51个农贸市场和较大型超市食品安全规范化、标准化建设；开展职业技能晋升培训3.37万人次，创业培训2673人，促进创业2777户，带动就业7102人；为1.62万名已婚农村贫困妇女免费提供“两癌”（乳腺癌、宫颈癌）筛查服务。十件民生实事全面完成。成功举办“爱我珠江”亲水节、清远首届马拉松赛等文化体育活动，群众性体育运动蓬勃发展。

（八）突出抓好体制改革，政府建设有新举措。着力打造“审批环节最少、效率最高、服务最优”的新型城市，被确定为全国八个相对集中行政许可权试点改革地区之一，也是全省唯一的地级试点城市。在粤东西北地区率先公布市级政府部门权责清单。制定县级政府行政审批事项通用目录，推进行政审批标准化建设。网上办事大厅提前实现市县镇村四级全覆盖，建设水平走在全省前列。全面完成县级“两建”信息平台建设，商改后续监管平台建成运行。全面实施“三证合一、一照一码”登记制度，全市新登记各类市场主体30474户，增长22%。全面落实新《预算法》。公务用车改革顺利完成。为每位副市长配备法律顾问。扎实开展“三严三实”专题教育，工作作风进一步转变。认真落实市人大及其常委会各项决定，主动接受市政协的民主监督，按期办理人大代表建议76件、政协提案234件。政务公开走向制度化，行政监察和审计监督得到强化，政府系统惩治和预防腐败体系不断健全，法治服务型政府建设迈上新台阶。

各位代表，过去一年全市经济社会发展成效明显，为“十二五”规划的实施划上了圆满句号。五年来，我们围绕改革、发展、稳定、民生主题，深入实施“桥头堡”发展战略，全面开启转型之路，更加注重发展质量，有效提升了科学发展水平，为新常态下的可持续发展奠定了良好基础。

五年来，我们着力转变发展方式，发展质量明显提升。坚持做优存量扩大增量，一手抓落后产能淘汰，一手抓现代产业发展。淘汰了全部立窑水泥产能，关停了粤连、粤阳火电厂，单位生产总值能耗累计下降21%；单位建设用地GDP产出提高35%；先进制造业和高技术制造业占工业比重分别上升0.6个和1个百分点，全市有高新技术企业76家，重点实验室、工程中心、工程实验室63家；茶叶等农业四大新支柱产业蓬勃发展；五年新增全国驰名商标9件、国家地理标志产品3个、省名牌产品39个、绿色食品51个、5A级旅游景区1个、4A级旅游景区7个；参与制（修）订国家标准15项、行业标准35项；举办了六次金政座谈会，五年新增各类金融机构29家。三次产业结构由2010年的13.7:45.6:40.7调整为2015年的14.8:38.9:46.3。

五年来，我们着力推动改革创新，发展活力不断增强。重点领域和关键环节改革深入推进，简政放权和职能转变步伐加快。在粤东西北地区率先出台商事改革后续监管负面清单、企业投资负面清单。推进“两集中、三到位”，市一级行政审批事项调整比率达54.4%，审批时限总体压缩56.7%。加强市场监管体系和信用体系建设，市场更加规范有序。积极推进投融资体制改革，探索“集装箱”并联审批和重点项目“直接落地”试点。完成市、县两级政府机构改革。推进社区网格化管理试点，改革社会组织管理制度。12345政府服务热线平台建成使用。

五年来，我们着力加快城乡建设，发展条件持续改善。扎实推进“五城同创”，成功创建为“国家园林城市”。大力实施“一心两核”城市扩容提质。燕湖新城骨干框架搭建基本完成，英德、连州新区建设全面启动，城市旧区改造全面铺开，各县城和中心镇面貌日新月异。累计完成交通基础设施投资360亿元，实现“县县通高速”，高速公路通车总里程居全省第二位。完成997公里国省道路面改造，全面消灭了市域内国省道“烂路”。等级公路总里程、公路密度居粤东西北首位。产业园区扩能增效实现突破，4个省级产业园区累计投入开发资金70.8亿元，共引进项目279个、总投资额726.6亿元，建成项目152个。建成高标准农田120万亩、美丽乡村277条。电网建设累计投入62亿元，新增变电容量418.6万千伏安。实现3G网络市域全覆盖，4G网络市县镇中心区域全覆盖。

五年来，我们着力推进协调发展，南北发展齐头并进。实施“南融北拓”发展战略，积极对接广州帮扶，推动实施帮扶和一体化项目530个，总投资1189亿元。加大北部地区支持力度，省、市累计转移支付北部地区资金224亿元，是“十一五”时期的1.6倍。主体功能区规划试点有序推进，县域经济实力明显增强。清城区发展平台更加优化，对接融入广州步伐加快。清新区加快融入大市区，发展条件明显改善。英德市园区经济蓬勃发展，成为全市工业发展的主战场。佛冈县主导产业优势突出，综合竞争力不断提升。连州市区域中心城市功能逐步增强，商贸物流日益繁荣。连山、连南两个自治县交通条件持续改善，民族品牌更加响亮。阳山县多项改革探索走在前列，农综改革、电商发展亮点纷呈。

五年来，我们着力加强环境整治，生态优势更加凸显。二氧化硫、氮氧化物、化学需氧量、氨氮分别下降27.3％、22.9%、15.8%和21.6%，全面完成“十二五”减排目标。落实属地行政首长“河长”责任制，全市污水处理能力增加到42.5万吨/天，完成了一批城市截污工程建设。累计淘汰“黄标车”3.5万台，推进陶瓷行业“煤改气”。开展强力治超和泥头车整治。完成“一县一场”建设，农村生活垃圾收运体系逐步形成。累计完成98.5万亩重点碳汇工程、902公里生态景观林带建设，建成乡村绿化美化示范村400条、森林公园41个、湿地公园9个，成为省林业生态市，生态优势更加凸显。

五年来，我们着力改善民生福祉，人民群众更得实惠。累计投入民生领域资金733亿元，是“十一五”时期的1.7倍。着力推进基本公共服务均等化，实现全国义务教育发展基本均衡县、省教育强县全覆盖，教学质量跃居粤东西北前列。医疗卫生服务水平不断提升，全民医保体系基本建立。公共文化体育事业蓬勃发展。新增城镇就业人数26.42万，失业再就业7.17万人。新建保障性住房9692套，棚户区改造5521套，华侨农场危房改造7185户。农网改造累计投入15.5亿元。圆满完成两轮扶贫开发“双到”工作任务，“两不具备”搬迁超额完成省定任务。食品药品安全保障能力有效提升，安全生产形势总体平稳。国防动员、人民武装、民族、宗教、外事、侨务、港澳、妇女儿童、老龄、残疾人、红十字、档案、人防、方志、气象等工作取得新成绩。

各位代表，“十二五”时期我市经济社会发展取得的良好成绩，是省委、省政府和市委正确领导的结果，是市人大及其常务委员会和市政协监督支持的结果，是全市人民同心同德、奋力开拓的结果，是港澳台同胞、海外侨胞和社会各界关心支持的结果，是兄弟城市尤其是广州对口帮扶的结果。在此，我代表市人民政府，向全市广大工人、农民、知识分子、干部职工、驻清解放军部队、武警消防官兵、人民警察和各民主党派、工商联、人民团体、社会各界人士表示崇高的敬意！向长期关心和支持清远建设的兄弟城市、香港和澳门特别行政区同胞、台湾同胞、海外侨胞和国际友人，表示衷心的感谢！

同时，我们也清醒认识到，我市经济社会发展还存在不少困难和问题：一是经济总量不大，产业层次不高，重大工业项目不多，创新能力亟待提升，加快转型发展依然任重道远。二是区域发展不协调问题突出，如何推动北部地区绿色崛起，缩小南北发展差距，还有待深化探索。三是生态环境保护压力加大，空气质量仍待改善，如何在加快发展的同时，保护好清远的绿水青山，还须付出更大努力。四是民生保障水平不高，优质公共服务资源不足，民生事业发展与全市人民的期盼仍有较大差距。五是政府服务效能有待提高，机关作风建设仍须加强，营商环境有待进一步优化。对于这些困难和问题，我们必须高度重视，采取切实有效措施加以解决。

科学谋划“十三五”发展 确保全面建成小康社会

“十三五”时期是我市南部地区加快融入珠三角、北部地区全面建成小康社会的决胜期。综合研判国内外形势，全球产业结构和国际经贸规则深度调整，新一轮科技革命和产业变革将给人们的生产生活方式带来深刻影响；我国经济发展步入新常态，经济增速、发展方式、产业结构、发展动力正在发生积极变化；我省粤东西北地区振兴发展战略和创新驱动发展战略全面实施，补齐粤东西北和贫困地区发展短板的力度加大；我市转型升级取得重大进展，广清一体化战略上升到省级层面。所有这些，都将给我们带来新的发展机遇。但同时，全球市场疲软、需求不足的态势短期内难以改变，经济下行压力依然较大，发展面临不少困难和问题。我们必须继续保持转型发展和振兴发展的战略定力，积极从改革创新中激活动力，从科技革命和产业变革中挖掘潜力，从区域合作中寻找助力，全力实现“南融北康”发展目标。

根据党的十八届五中全会、省委十一届五次全会精神和市委关于“十三五”规划的建议，市政府组织编制了《清远市国民经济和社会发展第十三个五年规划纲要（草案）》。《规划纲要（草案）》提出，“十三五”时期工作的总体要求是：高举中国特色社会主义伟大旗帜，坚持以邓小平理论、“三个代表”重要思想、科学发展观为指导，深入贯彻习近平总书记系列重要讲话精神，按照“四个全面”的战略布局，坚持发展第一要务，坚持创新、协调、绿色、开放、共享发展的基本理念，以全面建成小康社会为总目标，以提高发展质量和效益为中心，以实施南部地区加快融入珠三角和北部地区全面建成小康社会两个行动计划为抓手，深入实施广清一体化发展战略，加快形成引领经济发展新常态的体制机制和发展方式，统筹推进经济建设、政治建设、文化建设、社会建设和生态文明建设，确保如期全面建成小康社会。

“十三五”时期我市经济社会发展的主要目标是：经济保持中高速增长，生产总值年均增长10%左右，固定资产投资年均增长15%，地方一般公共预算收入年均增长9%，全社会消费品零售总额年均增长10%；新兴主导产业初步形成，先进制造业占规上工业增加值比重达到30%，全社会研发经费支出占生产总值比重达到1%，三次产业结构调整为8:49:43；常住人口城镇化率达到60%；城乡居民人均可支配收入年均增长高于经济增速，基本社会保险覆盖率为98%，每千常住人口执业（助理）医师达到2.8人，城镇登记失业率控制在3.5%以内；单位生产总值能耗、主要污染物排放完成省定任务，城镇生活污水集中处理率95%，城乡生活垃圾无害化处理率90%，森林覆盖率75%。

根据上述总体要求，实现“十三五”规划目标，必须做到“五个坚定不移”：一是坚定不移推动创新发展，力促产业转型升级。坚持把创新驱动作为振兴发展的重要抓手，强化企业创新主体地位，培育做强一批创新型企业。激发创新创业活力，推动大众创业、万众创新。实施“互联网+”行动计划，发展大数据产业，推动新技术、新产业、新业态蓬勃发展。办好国家级高新区。大力发展科技生态型农业，培育新型职业农民，建设一批农业电商基地、农业休闲旅游示范点、农产品加工与物流服务集聚区。构建产业新体系，建设以高性能金属材料为主导的新材料产业基地、汽车整车及关键零部件基地、电梯电气装备制造基地、生物医药与健康养生产业基地。加快发展现代服务业，建设主题公园集聚区、源潭物流园区、华南铁路物流产业集聚区。二是坚定不移推进广清一体化，力促区域协调发展。坚持区域协同、城乡一体发展。深入实施广清一体化战略，推动广清两地规划衔接、交通互联、产业互补、生态共建和社会融合，南部地区初步融入珠三角。加大对北部地区的扶持力度，指导北部地区将生态优势转化为经济优势，大力发展绿色能源、现代农业、生态旅游等产业。落实中央、省扶持少数民族地区发展有关政策，改善民族地区发展条件，发展民族特色优势产业，实现全市人民共同迈入小康社会。三是坚定不移守住环保底线，力促美丽清远建设。把绿色发展理念融入经济社会各个方面，推广应用节能低碳技术，促进资源节约高效利用，推动生产生活方式绿色低碳。加强环境保护和污染治理，推进大气、水、土壤污染修复，加大北江流域治理和林业生态建设力度，巩固清远生态优势。深入实施国家主体功能区建设试点示范总体方案，细化配套政策和绩效考核评价体系，推动“多规合一”和空间一张图管理，加快形成主体功能区格局。建立健全生态文明制度体系，积极创建全国文明城市、国家卫生城市、国家环保模范城市。四是坚定不移打造重点平台，力促对外开放合作。把握广清一体化、广佛肇+清云韶经济圈建设、粤湘桂合作、“一带一路”倡议实施的机遇，构建对内对外双向开放格局，打造开放合作新平台。按照大广州都市圈经济腹地的定位，重点建设广清产业园、广清园佛冈园区、广清旅游集聚区和空港经济区。探索建立粤湘桂农产品加工基地和贸易集散中心。强化省级产业转移园等载体建设，理顺招商引资引技引智体制机制，促进各县（市、区）联动、协同、错位招商。围绕产业链配套、供应链衔接、价值链增值，强化对重点地区、重点产业、重点企业、重点人才招商，引导外资和民资投向现代服务业、先进制造业和战略性新兴产业。五是坚定不移办好民生实事，力促人民共享成果。切实增加城乡居民收入，缩小收入差距，增强人民群众幸福感。完善社会保障体系，实施全民参保计划，着力提高保障水平。从解决人民群众最关心最直接最现实的利益问题入手，有效增加公共服务供给，大力提升公共服务水平。深入实施基本公共服务均等化，进一步提升北部地区和农村地区教育、医疗、文化、体育、养老服务水平。实施精准扶贫、精准脱贫，因人因地施策，提高扶贫实效。

各位代表，“十三五”发展的宏伟蓝图已经绘就，清远振兴发展的新航程已经开启，只要全市人民齐心协力、开拓进取，就一定能够开创更加幸福美好的明天！

扎实做好2016年工作 实现“十三五”发展开门红

2016年是“十三五”的开局之年，也是清远加快振兴发展的关键之年，做好今年的工作意义重大。今年政府工作的总体要求是：深入贯彻党的十八大和十八届三中、四中、五中全会以及中央经济工作会议、省委十一届五次、六次全会、市委六届十次全会精神，坚持振兴发展、转型发展不动摇；坚持稳中求进，狠抓改革攻坚，突出创新驱动，推动区域协调，提升开放水平，促进绿色发展，加强民生保障，努力为“十三五”科学发展开好局、起好步。

今年经济社会发展的主要预期目标是：生产总值增长8-8.5%；固定资产投资增长10%左右；地方一般公共预算收入增长8%；社会消费品零售总额增长10.5%；城乡居民收入增长高于生产总值增速；城镇登记失业率控制在3.5%以内，居民消费价格总指数控制在103.5左右；社会保障、资源环境保护、节能减排及安全生产等完成省定目标。

实现上述目标，必须抓好以下十方面工作：

（一）全面推进广清一体化。推动广清产业园加快建设，力促已签约项目落地，已落地项目投产，稳步开展扩园工作。启动广清产业园佛冈园区的开发建设。支持源潭物流园规划建设，积极争取纳入广州空港经济区范围。深入开展金融合作，力争10家以上中小企业到广州股权交易中心挂牌。推动碳排放权交易项目开发拓展。探索建立穗清农产品质量安全互认机制、供穗农产品“绿色通道”机制和信息共享、突发事件应急处理机制。推动公共服务共享。加快市人民医院二期建设，合作共建广州医科大学附属第六医院、第六临床学院。推动更多的广州名校到清远设立教育机构。出台政策积极引进广州人才、技术和创新团队。大力支持广州北江供水项目建设。认真落实北部地区全面建成小康社会行动计划年度工作。进一步加大对民族地区的政策、资金扶持力度，推动民族地区绿色发展。用好用足税收、用电等民族优惠政策，引导一批优质项目落户民族工业园。争取上级政策资金支持，加大市级投入，扶持北部地区发展一批绿色能源、现代农业、生态旅游项目。

（二）实施新一轮交通大建设。按照“五纵三横”高速公路网规划，推动汕昆、汕湛高速清远段、广清与清连高速连接线、二广与清连高速连接线、佛清从高速北段加快建设；加快连佛公路前期工作，力争年内动工。加快清远西部一级公路建设。做好二广高速连山至贺州支线前期工作。加快广清城际轨道建设，力争一期项目主体工程基本完工，北延线年内顺利动工，二期项目方案基本确定。做好“五位一体”交通枢纽规划设计。加快北江航道扩能升级步伐，推进国家大宗货物绿色运输北江示范项目建设，打造北江黄金水道。积极打通广清两市断头路，大力实施国省道与高速公路主要出口互联“对接路”改造提升，全面推进国省干线路域环境综合整治，完成清连二级公路生命防护“国家示范工程”建设，以及国道107线市区段等干线公路创建省级文明示范路建设。完成439公里农村公路建设和200公里县道烂路大修工程。

（三）扎实推进城市扩容提质。加快东门塘环境综合整治工程、湖城大道等在建项目建设，新开工北江南岸公园、松苏岭公园一期、大燕湖防灾减灾综合整治工程、职教一路等项目，力促一批项目“2.28”动工建设。加快北江四桥、五桥项目建设。注重城市特色风貌塑造。加强城市地下管网建设和地下空间利用。推动汽车充电桩规划建设。开工建设江南水厂一期、220千伏岭塘等供水供电项目，确保清新太和第二水厂扩建工程、南部供水管网扩建工程、220千伏保城2号主变扩建等供水供电项目按期投产。进一步完善公共交通、停车场等市政配套设施。加大信息基础设施投资规模，建成覆盖全市城乡的高速4G网络。完善房地产去库存政策。加强施工管理，避免施工扰民。深入开展创文、创卫活动，认真落实“门前三包”责任制，进一步提高城市管理水平。加快英德、连州新城建设。下大力气抓好镇村规划。

（四）推动创新发展和产业升级。以国家级高新区为平台，加大资金、政策支持力度，实施“扬帆”人才计划，力促企业创新发展。推动华南863、天安智谷等科技企业孵化器加快建设，扶持国家火炬清远高性能结构材料特色产业基地、新材料协同创新研究院和中试加速基地发展。引导企业以项目联合攻关、共建研发机构等形式开展产学研合作，加快高校、科研院所优秀科技成果在清远转化。加大政策落实力度，服务引导企业加强知识产权建设，进一步增加企业发明专利申请量和授权量。深入实施标准化战略。实施工业转型升级攻坚战三年行动计划，力争完成80个以上的技改项目，完成技改投资33亿元以上。进一步完善重点园区路网、水电、排污、通信等基础设施和生活服务类设施配套，支持物流、质量检测、科技信息服务等生产性服务业发展，提高产业承接能力，力争省级园区完成工业项目固定资产投资90亿元以上，规上工业增加值130亿元以上。加快处置“僵尸企业”，推动陶瓷、有色金属等传统产业“腾笼换鸟”，推进拆解遗留场地综合利用。深入开展质量强市活动，推进“首席质量官”试点，提升产品供给质量。打好降成本“组合拳”，落实减税降费、社保费率调整等政策，加快电力、流通等领域改革，降低企业运营成本。

（五）促进旅游产业集聚发展。举全市之力推动长隆项目加快建设，切实解决好用地、立项、道路交通、林地占用、动植物种源引进等问题。适应长隆项目建设，及时调整旅游发展规划，完善旅游产业集聚区布局。发挥长隆项目辐射带动效应，有针对性地设计旅游线路，配套开发健康养生、运动休闲、乡村生态游等多样化旅游产品。创新思维推进英西峰林等一批特色旅游小镇规划建设。加大智慧旅游信息平台建设，创新旅游营销方式。深化区域旅游合作，共同设计“一程多站”精品线路，打响广清一体和“华南五市”品牌。重塑国家女足训练基地，发挥恒大足球学校品牌优势，大力发展足球事业，推动足球产业与旅游业融合发展。抓好旅游市场整治，提高行业服务水平。

（六）加快商贸流通业发展。用好国家、省、市外贸稳增长政策，积极帮助企业开拓国际市场。推动外贸综合服务企业和跨境电商等外贸新业态发展。支持电子商务与快递业联动发展，加快跨境快递物流场地改造，争取早日投入使用。开启关检“三互”通关新模式，推行“一次申报、一次查验、一次放行”及口岸现场“单一窗口”通关改革，加快电子口岸建设，不断优化通关环境。推进“无水港”建设，积极对接广东自贸区。发挥万达广场、沃尔玛等项目的辐射带动效应，积极发展商贸服务业。以华南声谷、源潭物流园等为平台，做好现代物流、服务外包等新业态招商。加大资金投入，完善电商企业培育、人才引进培养、物流配送、金融服务、产品质量保障等体系建设，推动电子商务快速发展。

（七）深化“三农”综合改革。积极推进全国农村改革试验区各项试验任务，健全规范村级各种组织，推动村民自治有效落实，加快推进农村土地确权登记颁证，引导各地解决土地碎片化问题，继续探索整合财政涉农资金。抓好农村信用合作试点建设，加快农村信用信息数据库建设，试点推进宅基地、房屋等农村产权抵押融资，推广“政银保”合作经验，提升农村金融服务水平。加大农业基础设施投入，抓好高标准基本农田建设，推进省级水利建设示范县、中小河流治理等民生水利项目建设，补足农业发展“短板”。积极推进林农转型发展试点，大力发展林下经济。建立健全农技推广、农机服务、农业生产资料合作供销、农村金融、农村电商等五个服务体系，降低农业生产成本。积极培育种养大户、家庭农场、农民专业合作社，引领农户适应市场需求变化。利用“互联网+”和电商平台，降低农产品销售成本。建立健全农产品质量安全监管检测体系，加大重大动植物疫病防控力度。扩大政策性农业保险覆盖面。实施“农村环境综合整治工程”，统筹推进省卫生镇、卫生村创建活动和名镇名村示范村、美丽乡村建设。

（八）治理改善空气环境质量。积极发展绿色交通，进一步扩大“黄标车”限行区，加快淘汰“黄标车”、“老旧车”。扩大高污染燃料禁燃区，加强燃煤锅炉整治。做好强制性清洁生产审核，推进VOCs重点企业整治以及工业企业烟粉尘污染防治工作。加强建筑工地扬尘、道路扬尘、矿山扬尘、秸秆和垃圾焚烧等污染防治，进一步改善空气环境质量。抓好环保基础设施建设，推进清新与旧城污水处理厂二期工程及7座中心镇污水处理设施建设。狠抓工业污染防治，逐步取缔“十小”企业，加大力度整治“十大”企业。做好禽畜养殖场等农业面源污染控制，推进农村环境综合整治。加大城市黑臭水体整治力度，继续推进大燕河、乐排河等重点流域水污染防治工作。巩固电子废弃物整治成果，加快清理历史遗留废弃物残渣。争取完成全国环保模范城市创建各项任务。

（九）建设法治服务型政府。自觉接受人大及其常委会的工作监督、法律监督和政协的民主监督，定期向人大及其常委会报告工作，向政协通报情况，高质量办好人大代表建议和政协委员提案。做好地方立法的服务与配合工作。推进市、县、镇三级政府法律顾问全覆盖，推行重大行政决策风险评估、实施后评估、终身责任追究和责任倒查制度，提升行政决策科学化、民主化、法制化水平。依法有序全面履行政府职能，严格规范文明执法，强化对行政权力的制约和监督，着力解决信息公开不到位、规范性文件制定程序不规范等问题。组织实施相对集中行政许可权试点工作，探索建立权责清单管理制度，开展行政审批中介服务清理，优化提升网上办事大厅效率。深化乡镇行政体制改革，完善基层治理体系。推进公共资源交易平台体系改革。抓好国有林场改革工作。以“正风”行动、“公述民评”等活动为抓手，深入治理“庸懒散奢”和不作为、慢作为等问题，推进机关作风建设常态化。严格落实党风廉政建设责任制，强化行政监察和审计监督，推进廉洁政府建设。

（十）切实做好民生保障。统筹推进城乡就业和重点群体就业。加大职业培训力度，完善就业服务体系，提高劳动者就业能力。完善创业带动就业扶持政策，引导、鼓励和支持更多的劳动者就业创业。以非公有制企业从业人员、城镇个体工商户灵活就业人员和务工人员为重点，扩大社会保障覆盖面，努力实现“应保尽保”。实施精准扶贫三年攻坚，推动扶贫改革深入开展。完善社会救助体系。开展教育现代化先进市创建工作，抓好教育“十大工程”，更加注重内涵发展，不断提升教育发展水平。认真落实外来工子女就读政策。加快省级职教基地5所高职院校建设。启动卫生强市创建，深化医药卫生体制改革，积极扶持社会办医。坚持国家计生政策不动摇，全面落实一对夫妇可生育两孩政策。大力实施文化惠民工程，办好群众性文体活动，推进公共文化服务标准化建设。开工建设图书馆新馆、城市馆（博物馆）、科技馆、艺术中心等市级文化体育综合体。广泛开展全民健身活动，启动奥林匹克运动中心规划设计。抓好食品药品安全和生产安全，做好打假、打传、打私工作，积极预防和有效化解社会矛盾，推进平安清远建设。加强统计、民族、宗教、外事、侨务、港澳、人防、国防动员、档案、方志、气象、水文、慈善、妇女儿童、残疾人等工作。

各位代表，在落实省十件民生实事有关任务的同时，市政府将投入3.68亿元，全力办好今年十件民生实事：一是提高困难群体生活补助标准；二是全市城镇新增4万人就业；三是建设保障性安居工程6137套（户）；四是建设开放100个运动场馆；五是改造建设12151套农村危房；六是市区县城改造100条内街小巷；七是解决10万名以上农村居民的自来水供应问题；八是改善基层医疗卫生服务；九是创建100条以上的美丽乡村；十是中心城区新建10公里以上绿道。

各位代表，加快融入珠三角，全面建成小康社会，是时代赋予我们的光荣使命。让我们紧密团结在以习近平同志为总书记的党中央周围，按照省委、省政府和市委的决策部署，抢抓机遇，锐意进取，真抓实干，为建设富裕文明美丽清远而努力奋斗!
